# Semantic Path-Guided Remote Sensing Recommendation for Natural Disasters Based on Knowledge Graph

**DOI:** 10.3390/s25175575

**Published:** 2025-09-06

**Authors:** Xiangyu Zhao, Chunju Zhang, Chenchen Luo, Jun Zhang, Chaoqun Chu, Chenxi Li, Yifan Pei, Zhaofu Wu

**Affiliations:** 1 College of Civil Engineering, Hefei University of Technology, Hefei 230009, China; 2023110780@mail.hfut.edu.cn (X.Z.); 2022110637@mail.hfut.edu.cn (C.L.); 2023110779@mail.hfut.edu.cn (C.C.); 2024111596@mail.hfut.edu.cn (C.L.); 2024110759@mail.hfut.edu.cn (Y.P.); wuzhaofu@hfut.edu.cn (Z.W.); 2 National Geomatics Center of China, Beijing 100830, China; junzhang@ngcc.cn

**Keywords:** natural disaster, remote sensing imagery, knowledge graph, data recommendation

## Abstract

To address the challenges of complex task matching, limited semantic representation, and low recommendation efficiency in remote sensing data acquisition for natural disasters, this study proposes a semantic path-guided recommendation method based on a knowledge graph framework. A disaster-oriented remote sensing knowledge graph is constructed by integrating entities such as disaster types, remote sensing tasks, observation requirements, sensors, and satellite platforms. High-order meta-paths with semantic closure are designed to model task–resource relationships structurally. A Meta-Path2Vec embedding mechanism is employed to learn vector representations of nodes through path-constrained random walks and Skip-Gram training, capturing implicit semantic correlations between tasks and sensors. Cosine similarity and a Top-K ranking strategy are then applied to perform intelligent task-driven sensor recommendation. Experiments on multiple disaster scenarios—such as floods, landslides, and wildfires—demonstrate the model’s high accuracy and robust stability. An interactive recommendation system is also developed, integrating data querying, model inference, and visual feedback, validating the method’s practicality and effectiveness in real-world applications. This work provides a theoretical foundation and practical solution for intelligent remote sensing data matching in disaster contexts.

## 1. Introduction

Remote sensing refers to the acquisition of information about the Earth’s surface and atmosphere without direct contact, typically via satellite or airborne sensors. As a core technology in disaster monitoring and environmental management, it enables multi-source observation across spatial, spectral, and temporal dimensions. Owing to its broad spatial coverage, high resolution, and frequent revisit cycles, remote sensing data have become indispensable in natural disaster analysis, supporting critical tasks such as disaster detection, dynamic change tracking, and post-event damage assessment [1,2,3]. However, the heterogeneity of data sources and the diversity of technical parameters pose significant challenges for users in selecting appropriate data for specific disaster scenarios [4]. This selection process not only requires the integration of spatial resolution, spectral bands, and acquisition timing [5] but also demands alignment between task semantics and sensor capabilities, resulting in operational complexity and increased cognitive burden. At present, remote sensing data acquisition still largely relies on expert knowledge [6] and keyword-based search methods [7], which lack systematic modeling and semantic representation of the relationships between task objectives and data characteristics [8]. These limitations hinder the development of intelligent retrieval and automated recommendation mechanisms, particularly in time-critical applications such as emergency response, where accuracy, interpretability, and rapid decision-making are essential [9].

In this context, developing a knowledge organization mechanism with semantic understanding and reasoning capabilities has become a crucial step toward improving the intelligence of remote sensing data recommendation systems [10]. As a knowledge representation framework that integrates ontology modeling, entity-relation structures, and semantic inference [11], knowledge graphs can systematically organize domain-specific entities, attributes, and relationships within a graph-based structure. This enables the representation of complex semantics and the discovery of inter-entity associations. In recent years, knowledge graphs have shown significant potential in domains such as education [12], healthcare [13], and agriculture [14], where they serve as foundational technologies for intelligent retrieval, personalized recommendation, and semantic question answering.

In the domains of remote sensing and natural disaster management, several studies have constructed knowledge graphs targeting specific disaster types—such as earthquakes, floods, and landslides—resulting in specialized graphs like flood and landslide knowledge graphs. Some researchers have further explored their use in remote sensing image interpretation and disaster recognition. For example, Zhang et al. summarized approaches to remote sensing knowledge graph construction and proposed a semantic network that integrates task types, sensor capabilities, and application scenarios to enhance information organization [15]. Zhou et al. developed a geoscientific knowledge graph with spatiotemporal reasoning capabilities to support cross-scale semantic fusion of remote sensing data [16]. Ji et al. provided a comprehensive review of knowledge graph construction and reasoning strategies, underscoring the value of path modeling in semantic abstraction [17]. Additional studies have explored knowledge graphs for semantic integration and visual reasoning of heterogeneous remote sensing data [18], fusing remote sensing imagery with social media for disaster estimation [19], enhancing the synergy of optical and InSAR data for landslide detection [20], and improving recognition performance by embedding knowledge graphs into deep learning models [21]. However, most of these efforts have concentrated on semantic modeling and retrieval, with limited attention to recommendation approaches specifically driven by knowledge graphs for remote sensing data selection.

Current knowledge graph-based recommendation methods are largely developed in general domains such as social media and e-commerce. These systems typically model users, items, and attributes to identify semantic associations between user preferences and item features [22,23]. Introducing knowledge graphs into the remote sensing domain offers a novel semantic paradigm that addresses the shortcomings of traditional rule-based and keyword-driven retrieval methods [24], facilitating a shift from passive content-based search to structured, task-driven intelligent recommendation [25]. Given the inherent complexity of matching heterogeneous data sources with task demands and observation parameters—particularly in time-sensitive disaster response scenarios—semantic interpretation and path-based reasoning become indispensable. Nevertheless, a unified framework that integrates task semantics, structural modeling, and reasoning mechanisms remains absent, limiting the depth and intelligence of knowledge graph applications in remote sensing data recommendation.

To address this gap, this study targets remote sensing data recommendation in natural disaster contexts by constructing a disaster-oriented knowledge graph that integrates key elements such as disaster types, task demands, spectral requirements, sensor parameters, and platform configurations. Recognizing the inefficiency of existing approaches in identifying optimal resources for specific disaster scenarios, we propose a semantic path-guided representation learning framework based on the concept of meta-paths [26,27]. These meta-paths are designed around task semantics to model structured semantic relationships among entities. We adopt the Meta-Path2Vec embedding method, combining path-guided random walks with embedding learning, followed by cosine similarity-based ranking to generate prioritized recommendations. This approach supports the computational modeling of semantic meta-paths within the knowledge graph structure, enhancing controllability, interpretability, and generalizability.

Accordingly, this paper presents a remote sensing data recommendation method that integrates knowledge graph modeling, semantic path-based embedding, and graph-based reasoning. Focusing on representative remote sensing tasks related to natural disasters, we construct a hierarchical semantic knowledge graph, design task-driven semantic paths, and implement reasoning mechanisms under path constraints using embedding-based models. This closed-loop framework—from task modeling to recommendation output—offers a novel solution for enhancing semantic organization and advancing the intelligence of remote sensing recommendation systems. By incorporating artificial intelligence into the data selection process, the proposed method reduces reliance on expert knowledge and enables intelligent matching between disaster-specific needs and sensor capabilities. Such approaches may support real-time, AI-driven systems capable of autonomously selecting appropriate data and generating disaster-specific thematic products, laying the groundwork for a new generation of adaptive and automated remote sensing applications.

## 2. Construction of a Natural Disaster-Oriented Knowledge Graph for Remote Sensing Data Recommendation

### 2.1. Knowledge System for Natural Disaster Remote Sensing Data Recommendation

**Figure 1:**
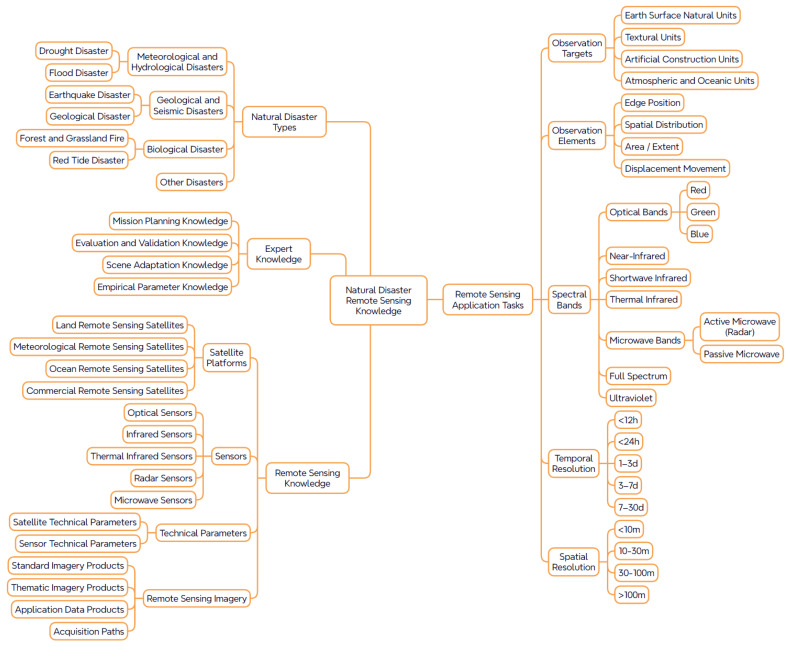
Semantic framework of the disaster knowledge graph for remote sensing data recommendation.

The intelligent recommendation of remote sensing data depends fundamentally on a clear understanding of monitoring requirements in natural disaster contexts, as well as the semantic alignment between task objectives and observational parameters. To support this process, the knowledge graph for remote sensing data recommendation in disaster scenarios must be built upon a structurally comprehensive knowledge system that enables logical progression from semantic modeling to path-based reasoning. This system consists of two core components: the first is a task knowledge system centered on natural disaster types and remote sensing application tasks, which defines the monitoring objectives and their associated observation parameters; the second is a remote sensing resource system that encapsulates the capabilities of satellite platforms, sensors, and data products, thereby reflecting the adaptability and responsiveness of available resources. Together, these two components form a semantic chain—“disaster type → remote sensing task demand → spectral band → remote sensing platform → image data”—as illustrated in Figure 1, which provides the foundational knowledge framework for reasoning and recommending remote sensing data.

Within this semantic chain, disaster types, remote sensing tasks, and spectral requirements function as the front-end semantic drivers, while platforms and sensors represent the responsive resource components. Through a path-constrained reasoning mechanism, the system enables intelligent task-to-resource matching, thereby supporting the efficient filtering and recommendation of remote sensing data.

#### 2.1.1. Knowledge System of Remote Sensing Application Tasks in Natural Disasters

Remote sensing application tasks constitute the foundational starting point for knowledge graph modeling. Variations in remote sensing response mechanisms across different disaster types result in distinct monitoring timeframes, spatial scales, and sensor configurations. Therefore, it is essential to construct a semantic recommendation chain that links disaster types to specific observational parameters, thereby capturing the mapping between task semantics and observational constraints. According to national standards GB/T 28921-2012 and GB/T 26376-2010 [28,29], natural disasters are classified into four primary categories: meteorological and hydrological disasters, geological and seismic disasters, marine disasters, and biological disasters. Typical events such as floods, landslides, and typhoons are subsumed under these classifications.

**Figure 2:**
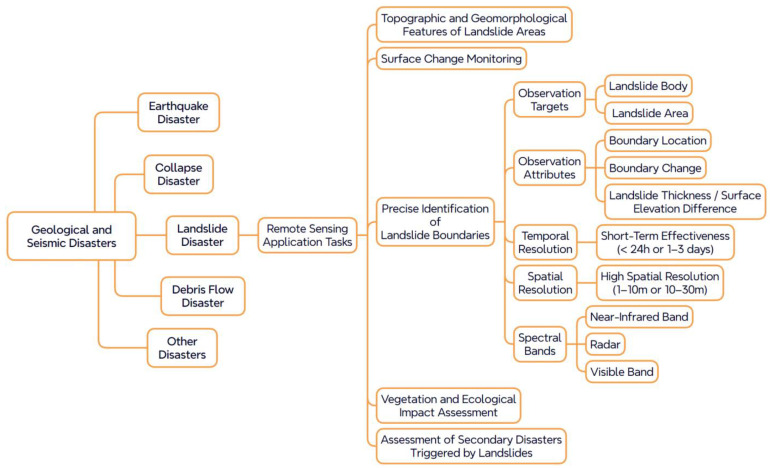
Example of remote sensing tasks and observation parameters for landslide disasters.

To ensure applicability within the context of remote sensing recommendation, this study selects disaster types that are both responsive to remote sensing and of high research relevance—namely, landslides, earthquakes, floods, droughts, and wildfires. Based on their characteristic monitoring requirements, task nodes are defined to represent specific objectives such as early warning, real-time monitoring, and post-disaster assessment. These are further decomposed into subtasks, including object recognition, boundary delineation, and deformation monitoring, thereby establishing a structured representation of task requirements and parameters, as illustrated in Figure 2.

To more systematically present the relationship between task requirements and associated parameters, Table 1 summarizes the observational demands of remote sensing tasks for three representative disaster types. These demands are categorized along five key dimensions: observed objects, observed attributes, temporal resolution, spatial resolution, and spectral bands. For example, flood-related tasks such as “water body detection” typically require optical or near-infrared imagery with a spatial resolution of 10–100 m and acquisition within 24 h, whereas earthquake-related tasks like “fault tracing” demand sub-meter spatial resolution and thermal infrared sensing capabilities. The tabulated information offers a clear overview of the response capabilities required for each task, laying a parameter foundation for subsequent resource modeling and semantic path reasoning.

Through this structured modeling approach, the knowledge system of remote sensing tasks in natural disaster contexts not only defines the semantic path’s origin but also establishes a logical and interpretable mapping from task requirements to observational parameters. This provides precise semantic constraints that are essential for intelligent remote sensing data recommendation.

#### 2.1.2. Remote Sensing Resource Knowledge System

**Figure 3:**
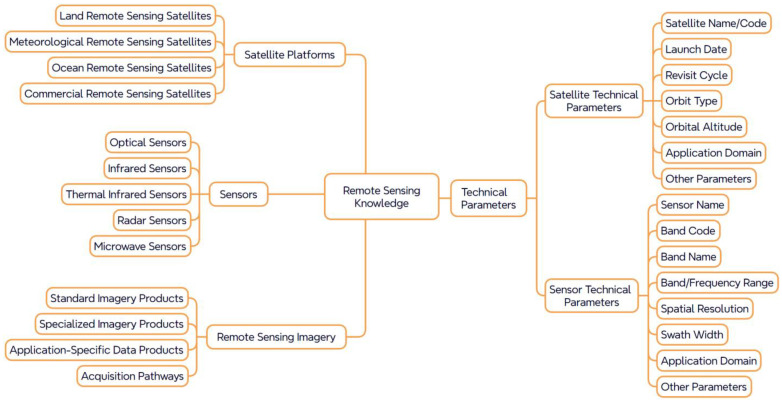
Framework of the Remote Sensing Satellite Knowledge System.

The remote sensing resource knowledge system serves as the responsive component within the semantic path reasoning chain, addressing the critical question: “What remote sensing capabilities are available?” This system is organized into four hierarchical layers—platform, sensor, parameter, and data product—as illustrated in Figure 3. Its objective is to construct a knowledge graph that accurately represents the observational capabilities of remote sensing systems, thereby providing the responsive foundation for a closed-loop semantic path connecting task demands with appropriate data resources.

At the platform level, remote sensing satellites are categorized based on their service objectives and operational contexts into land observation, meteorological, oceanic, and commercial platforms. Each category exhibits distinct capabilities in terms of orbital characteristics, revisit frequencies, and primary application domains. For instance, land observation satellites are typically employed for landslide monitoring and post-earthquake assessments, whereas meteorological satellites are better suited for wide-area flood and typhoon monitoring. As the core observational units of these platforms, sensors are classified into types such as optical, near-infrared, thermal infrared, radar, and microwave. Each sensor type supports specific disaster monitoring tasks depending on its imaging mechanism and spectral characteristics. For example, Synthetic Aperture Radar (SAR) is highly effective for monitoring ground deformation, while thermal infrared sensors are particularly suitable for wildfire detection due to their sensitivity to thermal anomalies.

In terms of capability representation, platform and sensor parameters play a pivotal role in task-to-resource matching. Platform parameters typically include basic attributes such as satellite name, orbital altitude, and revisit cycle. Sensor parameters, on the other hand, encompass technical specifications such as the number of spectral bands, spatial resolution, and swath width. These attributes serve as weighted indicators within the reasoning process, contributing to candidate filtering and recommendation ranking in the semantic path framework.

In summary, the remote sensing resource knowledge system in natural disaster scenarios establishes a structured linkage between task semantics and sensor capabilities. This linkage enables a closed-loop semantic representation from task definition to data recommendation. The system not only facilitates the construction of semantically coherent reasoning paths but also underpins the logic required by recommendation algorithms. Moreover, it provides essential knowledge support for the efficient and intelligent allocation of remote sensing resources in disaster-related applications.

### 2.2. Schema Layer Construction

At the core of the natural disaster-oriented remote sensing data recommendation system lies the structured organization and semantic modeling of heterogeneous domain knowledge. As the abstract design layer of the overall knowledge graph, the schema layer plays a pivotal role in ensuring the completeness of data representation and the viability of semantic reasoning. This section details the construction of the schema layer from three essential dimensions—concepts, attributes, and relationships—and proposes targeted semantic modeling strategies tailored to remote sensing application scenarios, thereby establishing both the structural and semantic foundation for downstream recommendation mechanisms.

In the conceptual modeling phase, this study organizes six major categories of semantic entities based on the task demands of remote sensing data recommendation: natural disaster types, remote sensing application tasks, observational parameters, sensors, satellite platforms, and remote sensing imagery. Among these, the entity type Disaster Type serves as the starting point of the graph structure and is further classified into five subcategories: meteorological and hydrological disasters, geological and seismic disasters, marine disasters, biological disasters, and other types. These are then refined into specific disaster events such as landslides, floods, and forest fires, forming a hierarchical taxonomy of disaster categories. The Remote Sensing Task (RS Task) acts as an intermediary semantic unit, bridging disaster types and sensing requirements. Its design fully considers variations across pre-disaster, during-disaster, and post-disaster phases, covering high-frequency modules such as terrain extraction, disaster change detection, and damage assessment, and is formalized into a structured task set.

To represent spectral requirements, two core semantic entities—Band Type and Spectral Range—are introduced. These entities express task dependencies on specific spectral categories (e.g., visible light, near-infrared, microwave) and quantify the required observational wavelength ranges, serving as semantic bridges between task nodes and sensing devices. Additionally, Sensor and Satellite entities are modeled from the perspectives of execution unit and platform support, respectively. Through abstraction and categorization of sensors (e.g., optical, radar) and satellite series (e.g., Gaofen, Sentinel), a clear entity classification system is established to support structured task–resource matching and scheduling.

**Figure 4:**
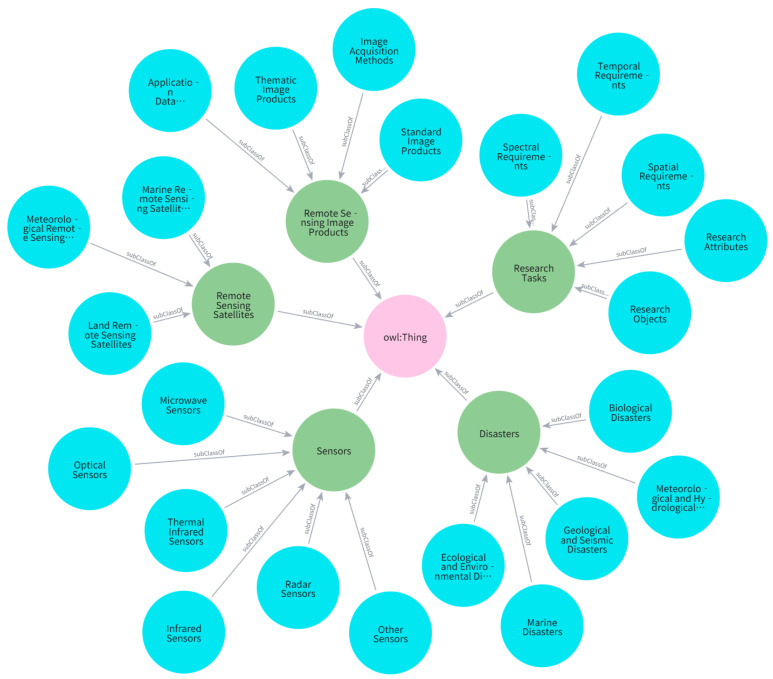
Ontological Structure of the Concept Layer.

Figure 4 illustrates the ontological framework of the conceptual system, in which hierarchical nodes capture the dependencies and associations among key semantic concepts, revealing the logical path from disaster recognition to data acquisition. This structure not only standardizes the granularity of entity classification but also defines the starting and terminal points of semantic path modeling. It demonstrates strong semantic closure and scalability, providing a stable knowledge foundation for path-constrained reasoning and embedding-based modeling.

**Figure 5:**
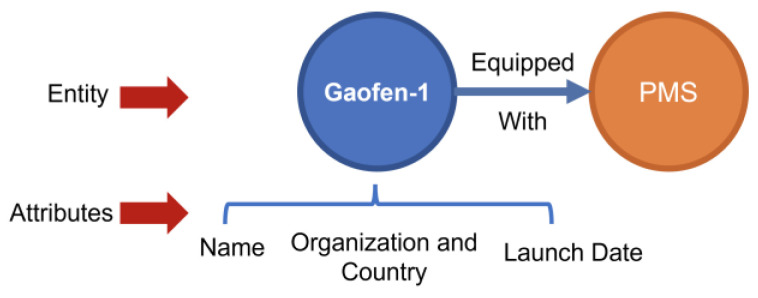
Illustration of Satellite Entity and Attributes.

In attribute modeling, to ensure the knowledge graph accurately reflects the technical characteristics and semantic constraints of each entity, this study defines a set of core attributes. For natural disasters, attributes include causal mechanisms, spatial distribution patterns, and response timeliness. For remote sensing tasks, attributes cover target categories, response stages, and observational dependencies. Sensor-related attributes include spectral type, spatial resolution, and application adaptability, while satellite platform attributes encompass orbital type, revisit cycle, and payload configuration. Figure 5 presents a typical attribute structure for satellite platforms, including fields such as country of origin, launch date, and equipped sensors, reflecting the multi-dimensional nature of remote sensing capabilities. Notably, certain attributes—such as spatial resolution and temporal resolution—can be abstracted as independent conceptual nodes, allowing them to directly participate in semantic path reasoning. This flexible transformation between attributes and concepts enhances the expressiveness and richness of semantic paths within the graph.

At the relationship modeling level, to represent the diverse semantic connections among entities, the system incorporates four fundamental relationship types: Hierarchy (HR), Is-A (ISA), Has-A, and Data Property. For example, Landslide as a subclass of Geological Disaster forms an HR relationship; Sentinel-2 as a specific satellite instance represents an ISA relationship; the OLI sensor having multiple bands such as Red and Green is expressed through a Has-A relation; and the spatial resolution of GF-1 WFV being 16 m is modeled via Data Property. In addition, a series of domain-specific functional relationships are defined to support path-based recommendation reasoning. These include ASSOCIATED_WITH (linking disaster types and tasks), REQUIRES_BAND (relating tasks to spectral requirements), and SPATTEMP_RES (associating tasks with spatial and temporal resolution constraints), which are semantically explicit and logically coherent. Other remote sensing-specific relations, such as EQUIPPED_WITH (linking satellites to onboard sensors) and CAPTURES (describing sensor-based data acquisition), further strengthen the logical foundation for matching task demands with sensing capabilities, thereby providing a computational basis for semantic path-guided recommendation reasoning.

### 2.3. Data Layer Construction

The construction of the data layer follows the structural blueprint established by the schema layer and is designed to generate instantiated knowledge content that supports semantic reasoning and recommendation computation. This is achieved through the acquisition, cleaning, extraction, and integration of multi-source data. The data layer not only provides the three fundamental components of a knowledge graph—entities, attributes, and relationships—but also demonstrates the feasibility of knowledge extraction techniques, the systematic organization of semantic information, and the practical alignment with real-world remote sensing task scenarios.

As summarized in Table 2, the data sources employed in this study span a variety of formats, including text documents, spreadsheets, and image metadata. These sources are categorized into four main types: (1) natural disaster event data, (2) satellite and sensor information, (3) remote sensing image metadata, and (4) auxiliary background materials. Among them, disaster event data serve as essential inputs for constructing the semantic chain of “disaster–task demand,” providing key parameters such as disaster type, occurrence time, affected area, and observation requirements. These data are primarily obtained from national emergency reports, disaster yearbooks, and official standards. Satellite and sensor information form the structural core of the “sensor–platform–imagery” linkage and are mainly sourced from technical manuals and databases maintained by organizations such as NASA, ESA, and CNSA. This category includes parameters related to platforms, sensor types, imaging modes, and spectral coverage, which collectively serve as the foundation for task–resource matching. Furthermore, remote sensing image metadata—focusing on aspects such as acquisition time, central coordinates, and spectral characteristics—describe the actual targets of recommendation and are sourced from open-access platforms such as the Geospatial Data Cloud and the China Centre for Resources Satellite Data and Application. Finally, auxiliary background materials serve as contextual supplements, offering information on disaster scenarios, sensor applicability, and classification schemes for remote sensing applications, thereby enriching the semantic depth of the knowledge graph.

**Figure 6:**
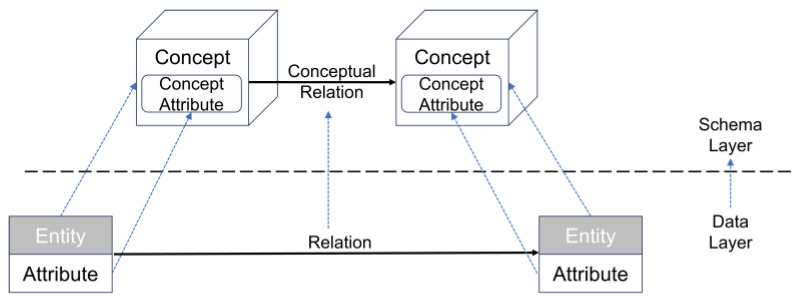
Mapping Between Schema Layer and Data Layer.

The construction of the data layer not only provides the material basis for instantiating the knowledge graph structure but also functions as a critical support mechanism for implementing the remote sensing data recommendation workflow. In this study, multi-source data are integrated and processed using a BiLSTM-CRF model [30,31,32] and semi-manual verification to extract key entities—such as disaster types, remote sensing tasks, satellite platforms, and sensors—from unstructured text. To ensure the accuracy and reliability of the constructed knowledge graph, the extracted results were carefully reviewed and corrected where necessary through human validation. Additionally, attribute information such as spatial resolution, spectral range, and imaging mode is extracted to enrich the graph’s semantic detail. The result is an instance-level remote sensing knowledge graph characterized by rich semantics, clear structure, and robust support for recommendation-related tasks. This comprehensive instance base provides a solid foundation for path-constrained reasoning and model training. Figure 6 illustrates the mapping between the schema and data layers: the upper section depicts the abstract conceptual framework, while the lower section shows the populated data instances—together forming a complete loop from structural definition to semantic realization.

To provide a clearer overview of the constructed knowledge graph, we summarize in Table 3 the number of nodes for the main entity types (e.g., disasters, tasks, platforms, sensors, and spectral bands), together with the counts of the core relationship types. This reflects the scale and structural composition of the knowledge graph used in this study.

### 2.4. Knowledge Graph Storage and Query

Graph databases offer superior performance in managing complex and densely connected structures, making them particularly well-suited for implementing knowledge graphs. This advantage is especially prominent in remote sensing applications, where entities often exhibit multi-level and multi-granularity interconnections. To efficiently manage the intricate semantic structures required for remote sensing data recommendation in disaster scenarios, this study adopts the Neo4j graph database for knowledge storage and query execution. Neo4j organizes data through nodes, relationships, and properties, making it a natural choice for modeling multi-relational semantic path structures such as “disaster type → remote sensing task demand → platform → data”. Additionally, Neo4j offers excellent scalability and query performance, making it ideal for large-scale semantic reasoning.

Queries on the knowledge graph are performed using Cypher, a declarative and intuitive graph query language that facilitates a range of operations including node attribute retrieval, path pattern matching, and relationship filtering. Neo4j also provides a robust programmable interface. For Python3.X users, the py2neo library enables seamless integration of the graph database with machine learning-based recommendation models, supporting efficient model training and inference while preserving the integrity of the graph structure.

**Figure 7:**
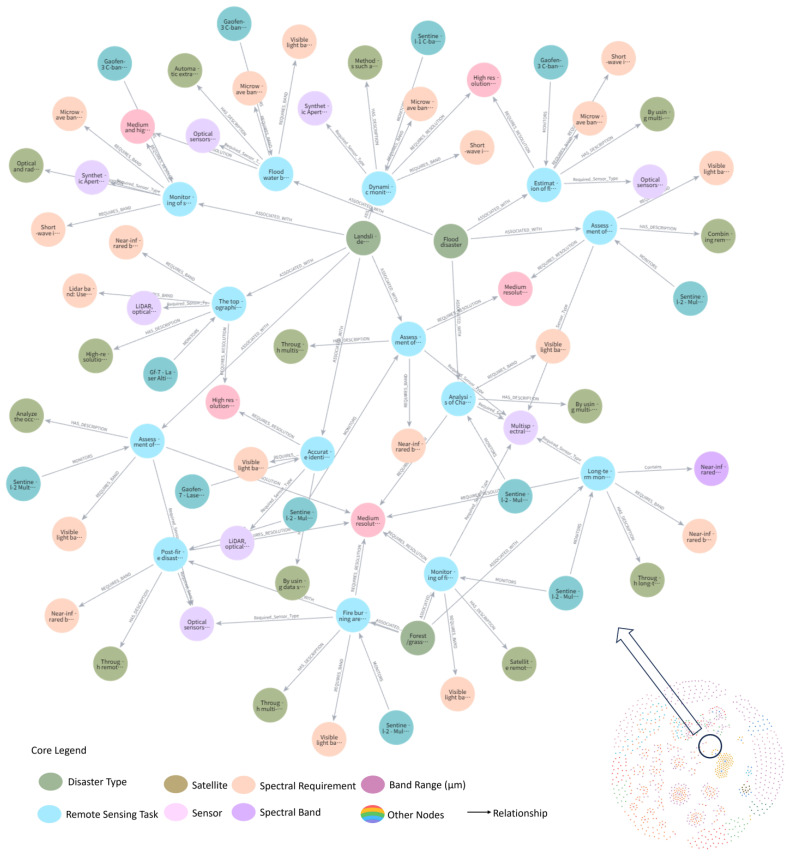
Knowledge Graph Visualization: Disaster Example.

Figure 7 presents a visualized subgraph of the knowledge graph for a flood disaster scenario. The central node, representing “flood disaster,” is connected via semantic relationships such as ASSOCIATED_WITH to nodes denoting relevant remote sensing tasks, required spectral band types for water body extraction, and spatial resolution parameters. This forms a semantically complete and logically coherent path network.

**Figure 8:**
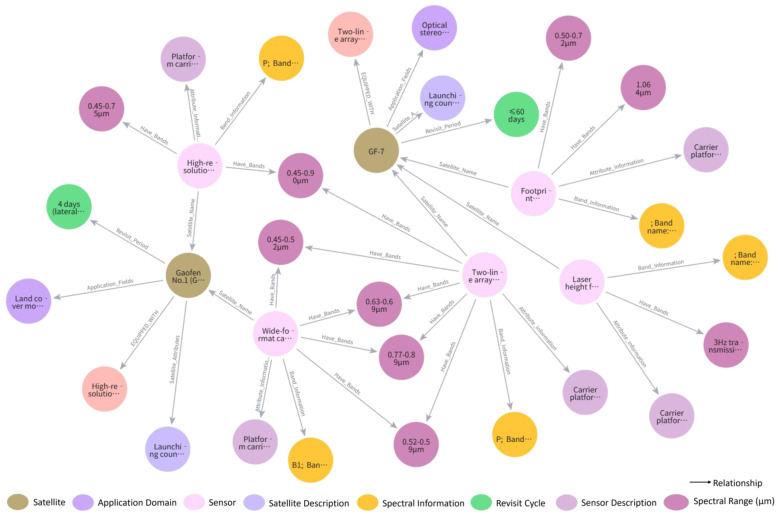
Knowledge Graph Visualization: Satellite and Sensor Example.

Figure 8 further illustrates the platform and sensor modules of the graph. Satellite platforms such as Gaofen-1 and Gaofen-6 are connected to onboard sensors like WFV (Wide Field of View) and PMS (Push-broom Multispectral Scanner), along with their associated spectral characteristics. Edges such as HAS_BAND and BELONGS_TO describe key attributes related to sensing capabilities and imaging modes. The interactive interface for the knowledge graph supports not only structural visualization but also dynamic exploration of upstream and downstream nodes and semantic paths. When combined with Cypher queries, this functionality allows for rapid knowledge validation and context-specific information retrieval.

In summary, the storage and querying capabilities provided by Neo4j ensure efficient, consistent, and scalable data management, while also delivering stable support for semantic modeling, feature acquisition, and reasoning-based remote sensing data recommendation. These functions make it a foundational component of the system architecture developed in this study.

## 3. Remote Sensing Data Recommendation Method Guided by Knowledge Graph Semantic Paths

Remote sensing tasks often involve heterogeneous information encompassing disaster types, observation targets, sensor capabilities, and platform configurations. Traditional recommendation approaches that rely solely on attribute similarity or adjacency-based structures often fail to capture the deep semantic relationships across different types of nodes. As such, developing a recommendation mechanism that integrates structural path reasoning with semantic constraints is essential for improving task-to-resource matching efficiency and enhancing the overall intelligence of remote sensing recommendation systems.

To enable structured semantic recommendation, this study proposes a knowledge graph semantic path-guided remote sensing data recommendation method. By designing meta-paths that encode high-order semantic constraints and employing the Meta-Path2Vec method [26,27] for node embedding, the model learns task–resource similarities within the embedding space. A Top-K ranking strategy is then applied to generate prioritized recommendation results. This end-to-end pipeline—from semantic modeling to embedding-based representation and reasoning-driven inference—provides both theoretical and practical support for achieving accurate, interpretable, and scalable remote sensing resource recommendation across diverse disaster types and task scenarios.

### 3.1. Semantic Path Modeling and Meta-Path Design Strategy

In remote sensing data recommendation, accurately matching task demands with sensor capabilities is of fundamental importance. To achieve this, we design a core meta-path grounded in the previously constructed natural disaster remote sensing knowledge graph. This meta-path is characterized by both structural connectivity and semantic coherence. Starting from the node representing the remote sensing task demand, the path sequentially connects nodes related to disaster type, spectral response characteristics, sensor capabilities, and satellite platforms. By modeling semantics at the path level, this approach significantly enhances both the accuracy and interpretability of the recommendation reasoning process.

**Figure 9:**
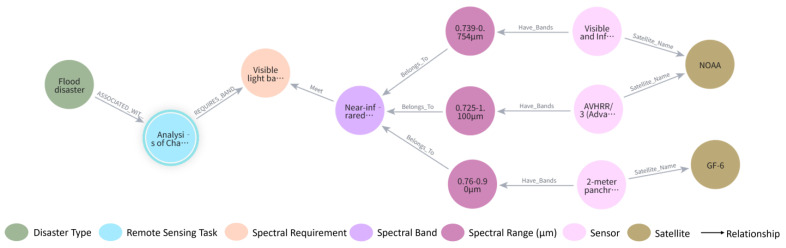
Example of a Meta-Path Structure.

To effectively capture the heterogeneous spectral requirements of remote sensing tasks, the meta-path structure is constructed with a focus on spectral response capacity as its primary semantic axis. While spatial and temporal resolution parameters remain critical in determining the overall usability of remote sensing data, their relationship with task performance is often more straightforward, as higher resolution generally contributes positively but does not always yield proportionally improved outcomes. In contrast, spectral information plays a more nuanced and task-specific role. For instance, flood detection benefits significantly from near-infrared bands due to the strong absorption characteristics of water, whereas wildfire monitoring relies heavily on mid-wave and thermal infrared bands to identify thermal anomalies. Compared with spatial or temporal resolution parameters, spectral information is inherently more structured and can be explicitly represented as nodes within the knowledge graph, thereby improving the semantic expressiveness of path transitions. For instance, in earthquake scenarios, boundary recognition tasks typically rely on high-resolution visible bands, while fire monitoring tasks prioritize mid-wave and thermal infrared bands to detect temperature anomalies. Figure 9 presents a sample meta-path structure for the task “Analysis of Changes Before and After Floods,” clearly illustrating the critical role of near-infrared bands in distinguishing water bodies from surrounding vegetation. Although different sensors may vary in their spectral response ranges, many remain suitable candidates within the same spectral category, and are thus included in the recommendation scope.

In designing the meta-path structure, we emphasize semantic directionality and transition stability, avoiding semantic noise caused by overly long paths or loosely connected relationships. Table 4 summarizes the node types and relationships involved in the designed meta-path. The path begins at a Disaster_Type node, proceeds through RS_Task, Spectral_Band, Band_Type, and Spectral_Range, and ultimately reaches Sensor nodes that support the required spectral range, as well as their corresponding Satellite nodes. This design not only defines a clear semantic propagation route for task requirements but also provides a standardized template for path-constrained node embedding.

During path modeling, one-to-many mappings frequently occur—for example, a single task may correspond to multiple sensors that satisfy its spectral requirements. This reflects the diversity of available remote sensing resources and underscores the need for path constraints and random walk mechanisms, which enhance both the flexibility and generalization capability of the recommendation model.

In conclusion, the proposed semantic path strategy—centered on spectral bands—achieves both structural convergence and semantic consistency. It provides a solid semantic foundation for guiding subsequent embedding learning and reasoning processes, thereby supporting effective and interpretable task-to-resource matching in remote sensing data recommendation.

### 3.2. Path-Guided Node Representation Learning Mechanism

To overcome the limitations of traditional embedding methods in capturing high-order semantic relationships among heterogeneous entity types, this study introduces a path-guided embedding mechanism. Predefined meta-paths are used as semantic structural constraints, systematically guiding the generation of node vector representations. Based on the Meta-Path2Vec model, the mechanism integrates random walk sampling strategies with a Skip-Gram-based Word2Vec training framework [33,34], resulting in a node embedding system that is both structurally constrained and semantically coherent. This ensures that the learned embeddings effectively capture latent semantic relationships between remote sensing tasks and sensor resources.

The first stage of this embedding mechanism employs a random walk strategy constrained by the predefined semantic paths, with the aim of improving the semantic purity of the training corpus. Traditional random walks often encounter disruptions from irrelevant nodes in the graph, generating noisy sequences that fail to reflect the structural and semantic requirements of the recommendation task. To address this issue, we define a meta-path template—as shown in Equation (1)—and impose strict constraints on the types of nodes and edges at each step of the walk. This ensures that generated paths follow the underlying recommendation logic and form semantically closed sequences.

(1) D→T→R→B→W→S→P

The constrained random walk begins at a Disaster_Type node and sequentially traverses nodes representing Remote Sensing Task (*T*), Spectral Band (*R*), Band Type (*B*), Spectral Range (*W*), Sensor (*S*), and Satellite (*P*), thereby forming path sequences that align with the designed semantic schema. To further enhance sequence quality, we implement a rollback strategy, detailed in Table 5: when a target node is missing, a required relationship is undefined, or the path reaches a dead end, the walker backtracks to the previous node and attempts an alternative path. This mechanism helps prevent invalid sequences from entering the training corpus and degrading the model’s performance.

After generating high-quality semantic path samples, the process proceeds to the second stage: node embedding training. Each valid node sequence that conforms to the meta-path template is treated as a “semantic sentence,” with individual nodes functioning as “words” within a structured corpus. To capture the contextual dependencies among nodes along the semantic paths, we apply the Skip-Gram model from the Word2Vec framework. This model learns low-dimensional vector representations by maximizing the co-occurrence probability between a center node and its context nodes within a defined sliding window. As a result, the training not only preserves the high-order semantic structure encoded in the meta-paths but also naturally clusters nodes with similar semantic functions (e.g., sensors sharing comparable spectral capabilities) within the embedding space. The resulting node embeddings provide a robust foundation for subsequent similarity-based matching and remote sensing resource recommendation, facilitating intelligent and semantically informed alignment between task requirements and sensing capabilities.

**Figure 10:**
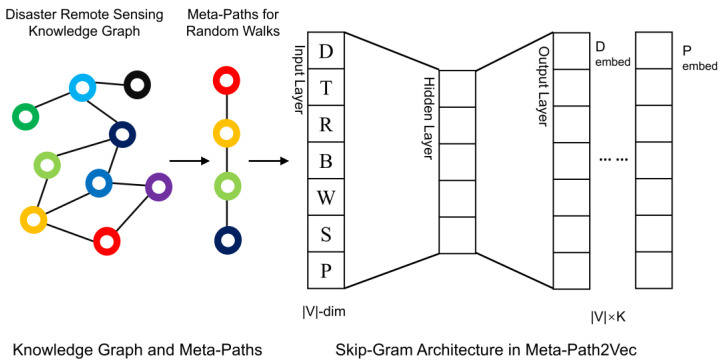
Meta-Path2Vec Training Based on Knowledge Graph.

Figure 10 illustrates the complete pipeline for node embedding learning. On the left is the constructed natural disaster knowledge graph; the middle section depicts the path-constrained random walk process; and the right side shows the Skip-Gram-based embedding training structure. In this workflow, the semantic paths and graph structure jointly constrain the learning process, ensuring both structural coherence and semantic expressiveness. Given the total number of nodes |V|, the embedding dimension is set to 128 to balance representational capacity with computational efficiency. The context window size determines the model’s ability to capture local structure along semantic paths.

**Figure 11:**
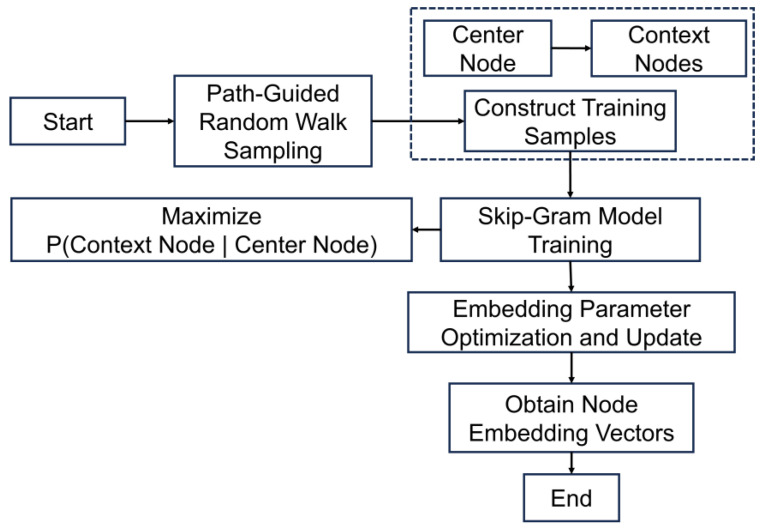
Skip-Gram Embedding Workflow Guided by Semantic Paths.

To further clarify the embedding workflow, Figure 11 provides a detailed breakdown of the Skip-Gram training process under meta-path guidance. Beginning with predefined meta-paths, semantic-constrained random walks are performed on the knowledge graph to generate node sequences relevant to specific remote sensing tasks. These sequences are then transformed into training samples by splitting each into center–context pairs using a sliding window. The Skip-Gram model is then used to optimize the conditional probability *P (context*∣*center)*, thereby learning node embeddings that reflect semantic co-occurrence patterns. After training, the resulting node vectors are employed to compute semantic similarity between task and sensor nodes, enabling Top-K recommendation for intelligent, task-driven remote sensing resource matching.

The proposed path-guided node representation learning mechanism effectively integrates the topological structure of the knowledge graph with the logical reasoning capabilities provided by semantic paths. By imposing meta-path constraints to ensure the quality of training data and applying Word2Vec-based embedding to capture latent semantic relationships, the mechanism produces embeddings that yield coherent and interpretable semantic representations of both remote sensing tasks and data resources. This not only enhances the explainability of recommendation results but also establishes a structured foundation for intelligent remote sensing data retrieval and resource matching.

### 3.3. Similarity Reasoning and Recommendation Output Mechanism

Upon completing the knowledge graph-based node embedding process, the critical next step is to transform the learned embeddings into sensor recommendation results tailored to specific remote sensing tasks. This section outlines the similarity computation and reasoning logic, introducing a Top-K ranking mechanism to produce the final recommendations. Together, these steps form a complete “embedding–reasoning–output” pipeline, enabling path-driven semantic recommendation. In this framework, the system calculates semantic similarity between task nodes and sensor nodes based on their embedding representations, using cosine similarity as the primary metric. A Top-K selection strategy is then applied to efficiently identify and rank the most relevant sensor candidates.

For similarity computation, cosine similarity [35,36] is employed as the core metric for measuring node proximity. Unlike Euclidean distance, cosine similarity is better suited for assessing the directional alignment of vectors in high-dimensional semantic space, making it particularly effective for capturing semantic closeness between vectors of different magnitudes. Specifically, each remote sensing task is represented by a composite embedding vector *u*, derived from both the disaster type and the associated remote sensing task node, while each sensor node is represented by a vector *v*. The semantic similarity between them is calculated using the cosine similarity function, as defined in Equation (2):

(2) $$\mathrm{Sim}(u,v)=\frac{u\cdot v}{\left||u||\cdot ||v|\right|}$$

where *u*·*v* denotes the dot product of the vectors, ||*u*|| and ||*v*|| represent their Euclidean norms. A similarity value closer to 1 indicates a higher degree of semantic consistency, and thus a stronger justification for recommendation. This semantic similarity-based mechanism allows the system to move beyond conventional rule-based approaches, adopting a more flexible and interpretable structure-aware recommendation paradigm.

Once similarity scores are computed for all candidate sensors, the system ranks them in descending order of similarity with respect to the target task node. The top *K* sensors are then selected as the final recommendation list. In this study, *K* is set to 4, striking a balance between comprehensive coverage of commonly used sensor resources and practical relevance in real-world applications.

It is worth emphasizing that while the meta-paths used in this study are primarily constructed based on spectral band requirements—to ensure alignment between recommended sensors and task-specific spectral needs—the underlying mechanism is inherently extensible. Additional constraints, such as temporal resolution and spatial resolution, can be readily incorporated in future iterations to further refine the precision of the recommendations under different task contexts. Ultimately, through the integration of semantic path modeling, embedding-based representation, and similarity reasoning, the proposed system achieves intelligent, interpretable, and task-driven sensor recommendation for remote sensing applications in disaster scenarios.

### 3.4. Experimental Data and Parameter Settings

The experimental dataset used in this study is derived from the previously constructed knowledge graph for natural disaster-oriented remote sensing data recommendation. This dataset encompasses multiple heterogeneous entity types—including disaster types, remote sensing tasks, spectral requirements, sensors, and satellite platforms—along with the semantic relationships linking them. It is specifically designed to support sensor recommendation tasks based on the Meta-Path2Vec embedding model, facilitating the learning of high-order semantic paths and the discovery of latent associations between task requirements and sensing resources. To ensure both robust model training and reliable inference results, the knowledge graph underwent systematic cleaning and processing, and all experiments were conducted in a high-performance computing environment.

During the data preprocessing phase, isolated nodes not connected to the target meta-paths were removed to ensure the structural integrity and semantic consistency of the embedding corpus. Subsequently, structured random walks were carried out based on predefined meta-path templates, generating node pair sequences used to train the Word2Vec model and learn vector-space representations. This process emphasized the modeling of co-occurrence relationships along semantic paths, resulting in node embeddings that simultaneously capture semantic coherence and discriminative power, thereby laying a solid foundation for the downstream recommendation process. Table 6 summarizes the key statistics of the experimental knowledge graph, which includes 872 nodes and 1208 relationships. A total of 500 structured walk sequences were generated under the guidance of the meta-path template to form the embedding corpus.

For node embedding implementation, the classical Word2Vec algorithm with a Skip-Gram architecture was employed to learn vector representations from the walk sequences. To enhance both model expressiveness and training efficiency, key hyperparameters were carefully configured, as summarized in Table 7. These parameter settings were not chosen arbitrarily; rather, they represent a combination of empirical experience from related studies and practical tuning on our dataset, where the current configuration provided a balanced trade-off between model performance and computational efficiency. The embedding dimension was set to 128, balancing representation capacity and computational cost. A context window size of 5 was chosen to capture the local neighborhood structure, while the number of negative samples was set to 10 to improve contrastive learning. The model was trained for 30 epochs, using an initial learning rate of 0.01 with exponential decay to ensure stable convergence. Additionally, to guarantee sufficient diversity and coverage of training samples, each node was sampled 50 times with a walk length of 7, producing high-quality semantic sequences for embedding learning.

Through the above data preparation pipeline and parameter configuration, this study successfully developed an embedding model characterized by strong semantic alignment and structural stability. The entire process—from semantic path design and structured sampling to model training and vector output—demonstrates methodological rigor and systematic implementation. This embedding system provides a reliable and interpretable foundation for the subsequent sensor recommendation algorithm and its comprehensive evaluation in disaster-related remote sensing applications.

## 4. Remote Sensing Data Recommendation: Validation and Analysis

### 4.1. Evaluation of Recommendation Results Based on Knowledge Graph Semantic Path Guidance

To evaluate the adaptability and effectiveness of the proposed semantic path embedding-based remote sensing data recommendation model across diverse disaster-related tasks, a task-driven evaluation framework was established. Drawing from remote sensing case studies on floods, landslides, and wildfires—collected from the China National Knowledge Infrastructure (CNKI)—a validation dataset comprising 14 distinct task types and a total of 240 instances was systematically curated. The dataset spans a broad spectrum of spectral combinations, ranging from visible to microwave bands, while also incorporating variations in spatial and temporal resolution requirements, thereby providing a comprehensive testbed for model validation.

A three-tier validation mechanism was adopted to assess the recommendation results, integrating (1) a literature-based reference library, (2) expert cross-validation, and (3) automated parameter verification. First, a standardized knowledge base was constructed to map remote sensing task demands to corresponding sensors, serving as a benchmark for evaluation. Second, domain experts in remote sensing were invited to manually assess the relevance and suitability of recommended sensors with respect to real-world task objectives. This expert review was supplemented by semantic retrieval and parameter-based verification techniques, ensuring the objectivity, thoroughness, and domain validity of the assessment process. The recommendation accuracy was calculated using Equation (3), defined as the proportion of valid recommendations within the model’s Top-K outputs.

(3) $$\mathrm{Overall}\;\mathrm{Accuracy}=\frac{\mathrm{Number}\;\mathrm{of}\;\mathrm{Valid}\;\mathrm{Recommendations}}{\mathrm{Total}\;\mathrm{Number}\;\mathrm{of}\;\mathrm{Top}\_K\;\mathrm{Recommendations}}\times 100\%$$

In this experimental evaluation, the model produced 56 sensor recommendations for 14 tasks spanning three disaster types. Among them, 31 recommendations were identified as valid, meaning they met the technical and semantic requirements of the corresponding tasks. This yields a task-level accuracy that reflects the model’s strong task-matching capability and cross-scenario adaptability. As shown in Table 8, the model performed particularly well for thermal infrared-related tasks, such as “fire detection and monitoring” and “fire intensity and behavior analysis”, both achieving 100% accuracy. In contrast, for tasks requiring high spatial resolution, such as “landslide boundary identification”, the model exhibited some deviation, resulting in lower accuracy. These results suggest that the proposed approach is particularly effective for thermal anomaly detection but requires further refinement for complex terrain interpretation.

Overall, the model’s performance remains stable and reliable when task definitions include explicit semantic paths and clearly defined spectral requirements. However, for tasks with incomplete metadata or those affected by multi-source interference, the recommendation accuracy tends to decrease. To address these limitations, future work may focus on enriching the semantic structure of the knowledge graph and incorporating dynamic constraints—such as real-time resolution thresholds or environmental conditions—to further enhance the precision, flexibility, and robustness of the recommendation system.

### 4.2. Stability Analysis of Remote Sensing Data Recommendation Based on Knowledge Graph Semantic Path Guidance

In practical applications, a recommendation system must exhibit robust stability, meaning it should consistently generate similar outputs under varying initialization conditions. To evaluate this aspect, we conducted a series of random seed experiments, using five regular seeds (10, 20, 30, 40, 50) and five irregular seeds (186, 360, 418, 718, 850), to comprehensively assess the model’s variability across different random initialization scenarios. For each seed, a unified semantic path embedding and recommendation process was applied, and results were evaluated accordingly. Among the tested seeds, Seed 850 demonstrated the best overall performance and was selected as the baseline for subsequent stability analysis.

To quantify the consistency of recommendation outputs across different seeds, we employed the Jaccard similarity metric [37,38] as the primary evaluation indicator. The corresponding formula is shown in Equation (4):

(4) $$\mathrm{Jaccard}(A,B)=\frac{\left|A\cap B\right|}{\left|A\cup B\right|}$$

where sets *A* and *B* represent the Top-K sensor recommendations for the same task under two different seed initializations. Jaccard similarity measures the ratio of intersection to union between two sets, making it especially suitable for evaluating the binary set overlap characteristic of recommendation outputs. To comprehensively assess model stability, we further calculated the mean Jaccard similarity (Jaccard Mean) and the variance of Jaccard similarity (Jaccard Variance) across all tasks and seed combinations. A higher Jaccard Mean indicates greater overall consistency, while a lower Jaccard Variance reflects reduced fluctuation between runs. Ideally, the Jaccard Mean should approach 1, and the Jaccard Variance should approach 0, indicating high stability.

**Figure 12:**
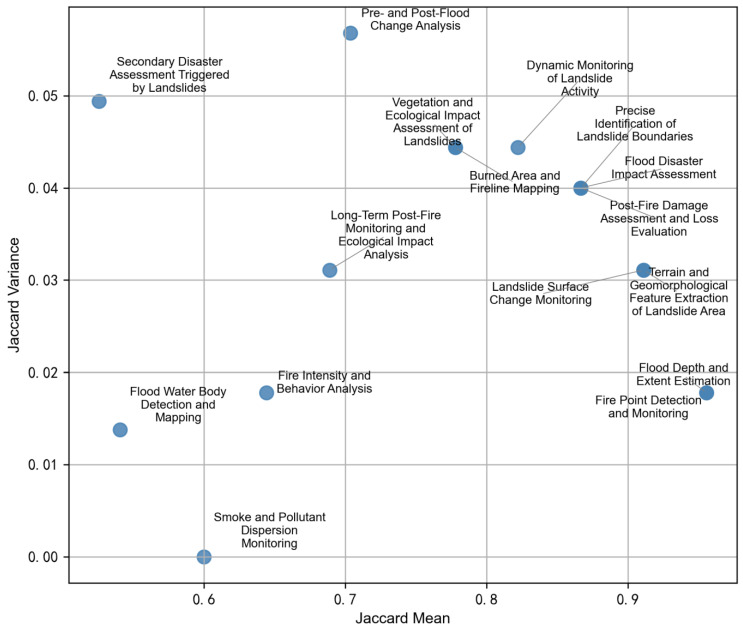
Stability Analysis of Remote Sensing Recommendation Tasks.

Figure 12 visualizes the stability results for different remote sensing tasks under Seed 850. The horizontal axis denotes Jaccard Mean, while the vertical axis represents Jaccard Variance. Tasks such as “fire detection and monitoring” and “terrain and geomorphological feature extraction in landslide areas” appear in the lower-right quadrant, indicating high consistency and low variance in the recommendations—demonstrating excellent model stability in these scenarios. In contrast, tasks like “assessment of secondary disasters from landslides” and “pre- and post-flood change analysis” are positioned in the upper-left quadrant, reflecting lower consistency and higher variability, possibly due to the semantic complexity or broader meta-path variability associated with these tasks.

To further evaluate the effectiveness of the proposed semantic path-based recommendation, we conducted a comparative experiment with a representative graph neural network (GNN)-based approach. Table 9 summarizes the matched counts across several disaster monitoring tasks. As shown, our method achieves more consistent alignment with task-specific requirements, especially in complex scenarios such as secondary disaster assessment and fire monitoring, where semantic interpretability plays a crucial role. In contrast, the GNN approach, while effective in some straightforward tasks, tends to underperform in cases requiring fine-grained semantic reasoning.

In addition to stability, computational efficiency is also a critical factor in evaluating the practical viability of recommendation models. To this end, we conducted a runtime comparison between the proposed semantic path-guided embedding model and a standard four-layer Graph Neural Network (GNN) model under identical computational environments. The experimental results indicate that the proposed model requires only 7.75 × 10^−3^ s per epoch on average, while the four-layer GNN model takes 8.89 × 10^−3^ s per epoch. Beyond runtime efficiency, the GNN baseline often produced fewer correct matches in complex disaster scenarios (see Table 9), particularly when task-specific spectral requirements had to be explicitly incorporated. In contrast, our semantic path-guided approach not only maintains computational efficiency but also achieves more reliable and interpretable recommendations. This balance of low training overhead and semantic accuracy makes the proposed method especially well-suited for real-time or near-real-time remote sensing data recommendation in time-sensitive disaster scenarios.

Overall, the model demonstrates strong stability for terrain-related and thermal anomaly related tasks, suggesting that the semantic path embedding strategy is particularly effective in these contexts. However, for more complex or ambiguous tasks, noticeable variations remain, pointing to a need for further optimization in meta-path design and feature constraint mechanisms. Enhancing the stability and generalizability of the system across diverse task scenarios will be a key focus for future improvements.

### 4.3. Remote Sensing Data Recommendation System

To facilitate efficient matching and intelligent acquisition of remote sensing data in natural disaster scenarios, this study develops a recommendation system grounded in a knowledge graph framework. The system adopts a browser–server (B/S) architecture with front-end/back-end separation, integrating knowledge graph querying, model inference, and visualization functionalities. This design ensures strong scalability, modularity, and interactivity. The overall system architecture is organized into three core functional modules: data processing, recommendation computation, and visual presentation. The data processing module handles user input, queries the Neo4j-based knowledge graph, extracts task-relevant subgraphs, and performs attribute-level preprocessing to ensure the semantic completeness and structural integrity of meta-paths required for recommendation. The visualization module dynamically displays the recommendation paths, outputs the Top-K sensor list, and presents associated metadata including similarity scores, image identifiers, and external data links, thereby supporting interactive query, semantic traceability, and result interpretability.

**Figure 13:**
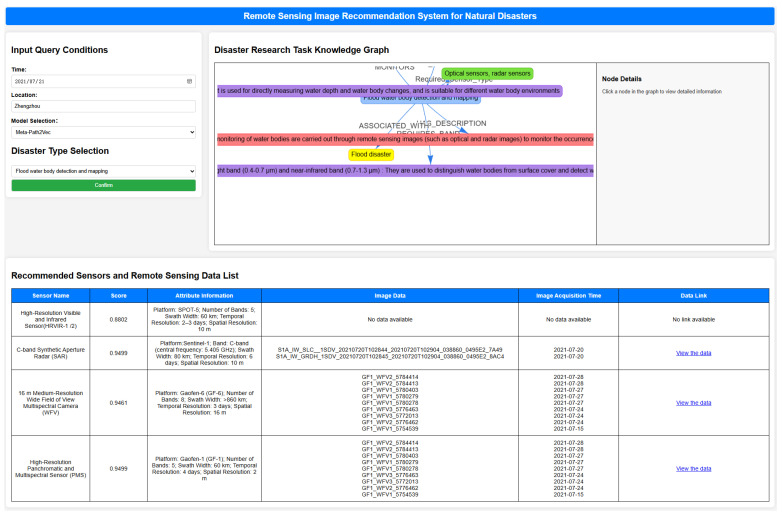
System Output Example.

To validate the system’s functionality, a real-world case study is employed: the Henan Zhengzhou flood event on 20 July 2021, with the corresponding remote sensing task defined as “Flood water body detection and mapping.” As illustrated in Figure 13, the system automatically generates the task-specific subgraph, invokes the recommendation model, and returns a Top-K list of sensor candidates with detailed attributes. The visualization interface displays a semantic path centered on the task node, linking it to the disaster type, spectral requirements, platform configurations, and resolution constraints, thereby forming a structured and semantically coherent representation that enhances user comprehension and semantic traceability.

As shown in the figure, the recommended sensors include Gaofen-1 WFV, Gaofen-1 PMS, and Sentinel-1 SAR, covering both optical and microwave modalities. Each sensor is annotated with its semantic similarity score, corresponding remote sensing image ID, acquisition time, and a direct download link, thus completing the loop from task input to data acquisition. The system’s visualization mechanism significantly improves the transparency, usability, and operational efficiency of the recommendation process, demonstrating the practical viability and application stability of the proposed framework in real-world disaster response scenarios.

## 5. Conclusions

This study addresses the challenge of intelligent remote sensing data recommendation in natural disaster scenarios by proposing a comprehensive methodological framework that integrates knowledge graph modeling, semantic path embedding, and recommendation reasoning mechanisms. A hierarchical disaster-oriented remote sensing knowledge graph was constructed to formally define the semantic chain of “natural disaster → task demand → sensor → remote sensing platform.” High-order meta-paths, designed based on task semantics, served as structural constraints in the Meta-Path2Vec-based node embedding process, enabling fine-grained semantic mapping between disaster monitoring tasks and observational resources. Through semantic similarity computation and Top-K ranking, the proposed framework achieves a closed-loop pipeline from semantic modeling to intelligent recommendation inference. Experimental evaluations demonstrate that the method achieves promising recommendation accuracy and stability across representative disaster scenarios, including flood monitoring, landslide detection, and wildfire assessment. Furthermore, system implementation confirms the feasibility, scalability, and practical value of the approach in real-world remote sensing applications, providing both a theoretical foundation and an engineering pathway for task-driven, intelligent matching of remote sensing resources.

Despite the effective integration of knowledge graph structures and path-based semantic embedding for remote sensing recommendation, the current system remains centered at the “data recommendation” level, primarily outputting raw imagery datasets. It has yet to incorporate higher-level thematic products, analysis-ready data, or automated processing workflows that are critical for end-to-end disaster response and decision-making. As a result, the system still falls short of supporting the full operational cycle from disaster onset to actionable information delivery. In addition, the recommendation accuracy is currently constrained by the static structure and limited semantic expressiveness of the knowledge graph, which hinders adaptability in handling diverse task types and uncertain or incomplete information. Future work should explore the integration of multi-dimensional constraints—such as temporal dynamics, spatial resolution requirements, and product-level metadata—alongside real-time graph updating mechanisms, to evolve the current architecture into a full-process intelligent service system spanning the chain of “disaster occurrence → data acquisition → product generation → knowledge application.” This shift—from data recommendation to content recommendation and workflow orchestration—would significantly elevate the intelligence, robustness, and applicability of the system, laying the groundwork for next-generation remote sensing recommendation services in complex disaster management scenarios.

## Figures and Tables

**Table 1:** Observation parameter examples for remote sensing tasks in typical natural disaster scenarios.

Disaster Type	Remote Sensing Task	Observed Object	Observed Attribute	Temporal Resolution	Spatial Resolution	Spectral Bands
Flood Disaster	Flood water detection and mapping	Flood extent	Water body boundary, expansion trend	<24 h	10–100 m	Visible, Near-infrared
	Estimation of flood depth and extent	Flood depth and boundaries	Water body thickness, height variation	<24 h	10–100 m	Thermal infrared, Radar
	Pre- and post-flood change analysis	Water body distribution	Water coverage changes	1–3 days	30–100 m	Visible, Near-infrared
	Flood impact assessment	Affected area	Disaster extent and distribution	3–7 days	30–100 m	Infrared, Radar
Earthquake	Surface deformation monitoring	Crustal displacement	3D deformation, displacement direction	<12 h	1–10 m	InSAR, LiDAR
	Crack and fault line tracing	Surface rupture zones	Crack direction, width, length	<24 h	0.3–5 m	Visible, Thermal infrared
	Damage assessment	Buildings and infrastructure	Collapse rate, degree of damage	1–3 days	0.5–3 m	Visible, Near-infrared, Radar
	Secondary disaster detection	Landslides/lakes/liquefaction	Scope and dynamics of secondary disasters	<24 h	10–30 m	Visible, Thermal infrared, Radar
	Post-disaster recovery and reconstruction	Rebuilding area	Land use change, engineering progress	Weeks–Months	1–30 m	Visible, Multi-temporal imagery
Forest/Grassland Fire	Fire point detection and monitoring	Hotspots and ignition areas	Thermal anomalies	<12 h	30–100 m	Thermal infrared
	Burned area and fireline mapping	Burned area boundaries	High-temperature regions	<24 h	10–30 m	Visible, Thermal infrared
	Fire intensity and behavior analysis	Fire dynamics	Fireline movement trajectories	<24 h	30–100 m	Visible, Thermal infrared
	Smoke and pollutant dispersion monitoring	Smoke and gas diffusion	Smoke concentration and dispersion trend	<24 h	30–500 m	Thermal infrared, Visible
	Post-fire assessment and damage evaluation	Burned zones	Burned area size	3–7 days	10–50 m	Infrared

**Table 2:** Data Sources.

Data Type	Format(s)	Data Content	Data Source
Natural Disaster Event Data	.txt; .xls; .pdf	Disaster type; occurrence time; affected area; impact status; observation parameters	Disaster monitoring reports; academic literature; national standards; historical disaster records
Satellite And Sensor Information	.csv; .json; .pdf; .xls	Satellite name; orbital parameters; observation frequency; onboard sensors; sensor specifications (spectral range, resolution, et al.)	Databases from space agencies such as NASA, ESA, CNSA; satellite manuals; technical documents; official websites
Remote Sensing Image Metadata	.csv; .mtl; .txt	Metadata of multispectral, thermal infrared, and SAR images; spatial and temporal resolution; spectral information; acquisition time	Geospatial Data Cloud; China Resources Satellite Data Center; NOAA; et al.
Auxiliary Background Materials	.txt; .xls; .pdf	Typical disaster case; disaster monitoring models; satellite applicability analysis; remote sensing classification standards	Remote sensing research papers; government environmental monitoring reports; international disaster databases

**Table 3:** Knowledge Graph Scale Statistics.

Category	Name	Count
Nodes	Disaster_Categories	4
	Research_Objective	85
	Spectral_Band_Requirement	22
	Band Type	17
	Spectral Range	195
	Sensor	36
	Satellite	20
Relations	ASSOCIATED_WITH	85
	REQUIRES_BAND	22
	MEET	80
	BELONGS_TO	191
	HAVE_BANDS	232
	EQUIPPED_WITH	20

**Table 4:** Meta-Path Node Types and Relationships.

Source Node Type	Relationship	Target Node Type
Disaster_Type (D)	ASSOCIATED_WITH	RS_Task (T)
RS_Task (T)	REQUIRES_BAND	Spectral_Band (R)
Band_Type (B)	MEET	Spectral_Band (R)
Spectral_Range (W)	BELONGS_TO	Band_Type (B)
Sensor (S)	HAVE_BANDS	Spectral_Range (W)
Satellite (P)	EQUIPPED_WITH	Sensor (S)

**Table 5:** Random Walk Rollback Strategy.

Encountered Issue	Action Taken
Target node does not exist	Backtrack to the previous node and select an alternative path
Relationship does not exist	Backtrack to the previous node and try a different relation
No available path	Mark as dead-end and discard the current path

**Table 6:** Statistics of Knowledge Graph Training Data.

Data Type	Quantity
Number of nodes acquired	872
Number of relationships acquired	1208
Number of walk paths generated	500

**Table 7:** Word2Vec Model Parameter Settings.

Parameter Name	Value	Description
Vector Dimension	128	Dimension of node embedding vectors; higher dimensions improve representation but increase computational cost
Window Size	5	Size of the Skip-Gram training window, determining the sampling range of neighboring nodes
Negative Samples	10	Number of negative samples, used to enhance training efficiency and embedding quality
Training Epochs	30	Number of training iterations to ensure sufficient model learning
Learning Rate	0.01	Initial learning rate with exponential decay to ensure stable convergence
Walk Length	7	Number of steps in each random walk, defining the length of the path
Walks per Node	50	Maximum number of random walks per starting node, ensuring adequate sample generation

**Table 8:** Validation Analysis of Recommendation Results (Examples).

Disaster Task	Matched Count	Task Accuracy	Evaluation Notes
Terrain and geomorphological feature extraction in landslide areas	3/4	75%	C-band multi-polarized SAR, LiDAR, and PMS (2 m panchromatic/8 m multispectral) are effective; MWR is less relevant.
Monitoring of landslide surface deformation	3/4	75%	LiDAR, C-band multi-polarized SAR, and C-band SAR are effective; SRAL is less effective.
Assessment of secondary disasters triggered by landslides	2/4	50%	16 m-resolution WFV and HRV-1/2 are effective; OLCI and stare cameras are less relevant.
Evaluation of vegetation and ecological impacts of landslides	2/4	50%	HRVIR-1/2 and PMS are effective; GMI and TROPOMI are less relevant.
Precise identification of landslide boundaries	1/4	25%	16 m-resolution WFV is effective; HRV-1/2, EMI, and NAOMI are less effective.
Fire intensity and behavior analysis	4/4	100%	TIRS, TIRS-2, AHSI, and C-band SAR are all core data sources.
Post-fire damage assessment and loss evaluation	2/4	50%	AVHRR/3 and MSI are effective; GMI and TROPOMI are less relevant.
Smoke and pollutant dispersion monitoring	2/4	50%	EMI and WFV are effective; stare cameras and NAOMI are less relevant.
Burned area and fireline mapping	1/4	25%	WFV and HRV-1/2 are effective; EMI and NAOMI are less relevant.
Fire point detection and monitoring	4/4	100%	TIRS-2, AHSI, TIRS, and ABI are all primary data sources.

**Table 9:** Comparative Results of Disaster Task Recommendations between the Proposed Semantic Path-Guided Model and the GNN Baseline.

Disaster Task	Matched Count (Proposed Model)	Matched Count (GNN)	Evaluation Notes
Terrain and geomorphological feature extraction in landslide areas	3/4	3/4	PMS, MSI, and OLI are effective; TROPOMI is less relevant.
Monitoring of landslide surface deformation	3/4	3/4	MSI, HRVIR-1/2, and OLI-2 are effective; AVHRR is less effective.
Assessment of secondary disasters triggered by landslides	2/4	1/4	OLI is effective; OLCI, GMI and DPC are less relevant.
Fire intensity and behavior analysis	4/4	2/4	C-band SAR (Sentinel-1 and GF-3) is effective; TIRS-2 and Laser altimeter are less relevant.
Post-fire damage assessment and loss evaluation	2/4	1/4	OLI is effective; OLCI, DPC and VIMS are less relevant.
Smoke and pollutant dispersion monitoring	2/4	2/4	SLSTR and OLI-2 are effective; VIIRS and ABI are less relevant.
Burned area and fireline mapping	1/4	2/4	SLSTR and OLI-2 are effective; VIIRS and ABI are less relevant.
Fire point detection and monitoring	4/4	2/4	TIRS-2, and ABI are effective; VIMS and AVHRR/3 are less relevant.

## Data Availability

Data could be shared upon request to the corresponding author.
